# Nutritional value of the marine fish in Bangladesh and their potential to address malnutrition: A review

**DOI:** 10.1016/j.heliyon.2023.e13385

**Published:** 2023-02-08

**Authors:** M.A. Rifat, Md. Abdul Wahab, Muhammad Arifur Rahman, Md. Nahiduzzaman, Abdullah-Al Mamun

**Affiliations:** a Department of Global Public Health, Karolinska Institutet, Stockholm, 171 77, Sweden; b WorldFish, Dhaka, 1212, Bangladesh; c Noakhali Science and Technology University, Noakhali, 3814, Bangladesh

**Keywords:** Marine fish, Pelagic fish, Small fish, Nutrient composition, Malnutrition, Small-scale fishery, Bangladesh, Lower middle-income countries

## Abstract

Marine fish are good source of essential macro- and micronutrients and major food items in coastal areas in Bangladesh. However, there is no review that details the nutritional value of marine fish in Bangladesh. Therefore, this review focuses on the nutrient composition of marine fish in Bangladesh and how the marine fish can address common nutrient deficiencies among women and children. Nutrient composition data was collected through literature searching in databases and source, including PubMed, Web of Science, Google Scholar, ScienceDirect, WorldFish, and Bangladesh-based database Banglajol. Calculation was carried out to present how one serving marine fish could potentially meet the daily requirement of protein, iron, zinc, calcium, vitamin A, and docosahexaenoic acid (DHA) for pregnant and lactating women and children aged 6–23 months. A total of 97 entries covering nutrient composition analysis of 67 individual fish species were extracted from 12 articles published between 1993 and 2020. Included articles contained analysis of proximate composition, vitamins, minerals, fatty acids, and amino acid. Twelve minerals and nine vitamins were analyzed and reported. The average energy, protein, fat, and ash content per 100 g edible raw marine fish was 343.58 kJ, 16.76 g, 4.16 g, and 2.22 g, respectively. According to available data, marine fish are good sources of protein, zinc, calcium, and DHA. Pelagic small fish, which are mainly captured by artisanal small-scale fishers, had more nutritional value than other categories of fish. Furthermore, marine small fish were found more nutritious than commonly consumed freshwater fish types in Bangladesh, including major carps, introduced carps, and tilapia. Therefore, the study concludes that marine fish have high potential to address malnutrition in Bangladesh. There was scarcity of literature regarding the nutrient composition of marine fish in Bangladesh and in South Asia as a whole, so more comprehensive quality research in this area is recommended.

## Highlights

• Marine fish are a good source of protein, Fe, Zn, Ca, vitamin A, and DHA.
• Pelagic small fish are more nutritious than other categories of fish.
• Marine fish could potentially address common nutrient deficiencies.
• Scarcity of research exists, therefore, more marine fish should be analyzed.
• Future research should emphasize on analysis of vitamins, minerals, and heavy metals.

## 1 Introduction

Globally, fish is the source of food and livelihood for more than one billion people [1], and an estimated >59 million people are involved in fishery production [2]. Most of the people involved in fishery production are from developing nations and are small-scale artisanal fishers [2]. Considering the importance of small-scale artisanal fishery, the Food and Agriculture Organization (FAO) of the United Nations declared 2022 as the “International Year of Artisanal Fisheries and Aquaculture 2022”. A major portion of the aquatic foods come from marine sources. Seafood consists of a variety of animals, plants, and microorganisms; however, fish contribute to the most significant share of the total seafood. Global total capture or fishery production from marine waters is more than seven times higher than that of inland water [1]. As one of the leading fish producing countries in the world, Bangladesh's fisheries sector receives a significant contribution from marine fishery, which covers an area of about 118,813 km^2^ along with 200 nautical miles of the exclusive economic zone from the coastline of Bay of Bengal [3]. In Bangladesh, the fisheries sector makes up 3.5% of the national Gross Domestic Product (GDP) and more than a quarter (25.72%) of the overall agricultural GDP [4]. About 270,000 fishing households in the coastal region somehow rely on marine fishery for livelihoods [5]. Marine fishery in Bangladesh consists of two subsectors [1]: the artisanal small-scale fishery, which makes up 83.75% of marine production and [2] the industrial fishery, which makes up the rest (16.25%) [4].

In Bangladesh, fish accounts for two-third of the total animal protein consumption. Considering per capita daily intake, fish (62.6 g) is the third-most consumed food— after starches (471.3 g) and vegetables (167.3 g)— covering approximately 60% of total annual animal protein intake [6,7]. Moreover, only fish intake exceeds the recommended daily amount (60 g) when compared with any other items in the food basket. There has been an increase in dietary consumption over the time [7]; however, malnutrition in Bangladesh is still a serious public health concern. Almost one-third (30.8%) of under-five children were found to suffer from chronic malnutrition or stunting, while half of pregnant women are anemic and more than half (57.3%) of non-pregnant and non-lactating (NPNL) women were zinc deficient [8,9]. On the other hand, there is an increasing trend of non-communicable diseases associated with overweight and obesity that indicates the double burden of malnutrition in Bangladesh [10,11]. Therefore, a shift from the quantity to quality dietary intake is important to tackle the high malnutrition burden. In that case, understanding the nutrient composition can be useful for the right food choice not only to improve diet quality but also to prevent nutrient-specific malnutrition.

Marine fish are rich sources of essential macro- and micronutrients which are highly bioavailable, easily digestible for all age groups, and beneficial to promote human health [12]. Fish are generally well known for containing highly bioavailable protein and good quality fatty acids, such as long-chain omega-3 polyunsaturated fatty acids. However, recent reports have highlighted that fish are also an important source of vitamins and minerals, such as vitamin D, selenium, zinc, phosphorus, and calcium [13,14]. Because of their high nutritional value, marine fish consumption is associated with many health benefits from fetal life to adulthood. These benefits include neurodevelopment at the embryonic stage, cognitive and visual development during infancy and childhood, and lowering the risk of cardiovascular diseases during adulthood [[15], [16], [17], [18], [19]].

Globally, data regarding the nutrient composition of fish is inadequate. The nutrition composition of only 526 fish species (25.7%) has been recorded in the Food and Agriculture Organization's INFOODS database, which is commonly used to calculate nutrient consumption, whereas a total of 2033 fish species are listed [1,20]. A similar scenario also exists for Bangladesh. Only 15 out of 475 identified marine fish species have had their nutrient composition recorded in the country's Food Composition Table [21,22]. Compounding the problem, policymakers are focused on the commercial importance of marine fish whereas nutritional values are usually overlooked [23]. As a result, because of the scarcity of nutritional data and the lack of policy attention, fish is often considered as a homogenous food group in dietary planning despite the remarkable nutrient content of many species that could potentially play a role in addressing specific nutrient deficiencies if promoted for consumption in adequate amounts [24]. For example, a promotion program for mola carplet (*Amblypharyngodon mola*), a small indigenous fish with high vitamin A content, would be a cost-effective approach to reduce the burden of micronutrient deficiency in Bangladesh [25].

This review examines the nutritional qualities of marine fish in the Bay of Bengal that are caught in Bangladesh marine territory. The findings could be useful in several ways. First, this review identifies marine fish with high nutritional value and assess their potential to address common nutrient deficiencies. Second, the findings can be useful to promote the consumption of nutrient-rich fish to enhance diet quality. Despite an increase in dietary fish consumption, micronutrient intake was still found to be decreased [26]. As a result, choosing fish with high nutritional value could potentially increase micronutrient intake. Third, the findings encourage the consumption of nutritionally rich marine fish among all socioeconomic groups. In last decade, Bangladesh has gone through positive socioeconomic changes; however, an increase in income and education was found to impart a status bias toward eggs and meat although they are comparatively more expensive and, in some cases, less beneficial than fish [27]. Finally, we have reported policy gaps and identified important fish species which have not yet been analyzed for their nutrient composition. This review could be useful to understand nutritional quality of marine fishes in Bangladesh required for dietary and livelihood related planning, marine policy formulation, and trade related purposes both national and international settings.

## 2 Methods

### 2.1 Data sources and search strategy

We conducted literature searching in five databases: PubMed, Web of Science, Google Scholar, ScienceDirect, and Bangladesh-based database Banglajol. The search strategy was as follows:

(fish* OR “sea fish” OR “pelagic fish” OR “marine fish” OR “small pelagic fish” OR “small marine fish”) AND (nutri* OR *nutri* composition OR composition OR “nutritional value” OR vitamin* OR mineral* OR carbohydrate OR protein OR fat OR “fatty acid” OR lipid* OR “amino acid”) AND (Bangladesh OR “Bay of Bengal” OR "Cox's Bazar").

In addition, we used keywords, including marine fish, pelagic fish, nutrient composition, Bangladesh, and Bay of Bengal, to conduct literature searching in Google Scholar, ScienceDirect, WorldFish, and Banglajol.

We did not apply any filter while conducting literature search in Web of Science and PubMed. To maximize the likelihood of getting relevant articles, reference lists of the included studies were also searched and examined “cited by” references in the Web of Science. Database searching was conducted from December 2020 to August 2021. Before conducting screening, all citations were imported into Mendeley reference manager, and duplication was checked.

### 2.2 Inclusion and exclusion criteria

The initial screening (title and abstract) criteria include [1]: The article/record is original research [2], The article/record contains the nutrient composition of marine fish, and [3] The article/record considers marine fish captured from Bangladesh water bodies.

Articles that qualified titles and abstracts screening were considered for full text screening. Several data extraction criteria were followed while full text screening. These included [1]: total number of fish species analyzed [2], local, English, and scientific names of the fish species [3], places of sample collection [4], number and types of nutrients considered for analysis [5], laboratory methods for nutrient analysis, and [6] statistical representation of the nutritional data. Furthermore, the following inclusion criteria were considered.

• Publication date: no specific time frame was considered.
• Language: English.
• Laboratory methods: no article was excluded due to the types of laboratory methods used to assess nutrient composition because some analytical methods represent better efficiency to detect specific nutrients and subtypes of some nutrients, such as different forms of vitamin A.
• Sample type: articles with nutrient composition of raw fish were considered. Articles representing the nutrient composition of dry fish, other marine aquatic species, cooked or processed seafood, and/or marine fish-based products were not included.

Articles that did not fulfill the research question, were still under review or were not available online (found after reference searching) were excluded with justification provided.

### 2.3 Selection process

According to agreed search strategies, two researchers independently conducted literature searching, including titles and abstracts screening and full-text screening. The variance in the number of relevant articles at every stage of screening was assessed by another reviewer. To minimize the bias, any discord during screening process was settled through discussion among the reviewers. Articles/records that qualified the full text screening and met the inclusion criteria were considered for data extraction and included in the review.

### 2.4 Data extraction

Data was extracted from the included articles independently by two reviewers. Microsoft Excel spreadsheet was used for data extraction. Data extraction considered the identity of the fish species (local, English, and scientific names) and nutrient content per 100 g of edible raw marine fish. Values were converted into nutrient content per 100 g edible raw marine fish if they were presented in nutrient content per 1 kg of weight or in other units, including ppm or percentage. If an article contained the nutrient composition of a wide range of fish species (marine and inland water), shrimps, and/or other aquatic animals, we only extracted data for marine fish and excluded others. Nutrient compositions are often represented as mean ± standard deviation (SD) while several samples were analyzed; however, SD values were not included during data extraction. Common and commercially important fish species are of high research interest and their nutrient compositions were reported in several articles. In those cases, all the findings/results were considered for data extraction allowing several entries of nutrient composition for a single species. For fatty acid and lipid compositions, total saturated fatty acid (SFA), monounsaturated fatty acid (MUFA), polyunsaturated fatty acid (PUFA), ecosapentaenoic acid (EPA), docosahexaenoic acid (DHA), and total cholesterol content were considered for data extraction. For missing values, no results, trace values, not detected, and unreported information (such as English name and local name of the species and type of tissue processing), cells were kept blank in the data extraction spreadsheet. While data extraction, no self-correction or changing the spelling of local name, English name, and scientific names of any fish species reported in the included articles was carried out (Supplementary file 1).

### 2.5 Potential contribution to address malnutrition

To observe potential contribution to address malnutrition, we focused on six nutrients, including protein, zinc, iron, calcium, vitamin A, and DHA, based on national and global nutrition situation and the nutritional uniqueness of marine fish. For example, 8% under-5 children in Bangladesh are wasted (weight for height below the standard) which could be attributable to low protein consumption [9]. Micronutrients, including zinc, iron, calcium, and vitamin A, were considered because these nutrients are deficient in the diet globally [28,29]. DHA was also considered given that this essential fatty acid is highly found in marine fish and was frequently analyzed.

For each of the six nutrients, a calculation was carried out to present how the nutrient content of a serving of raw fish could potentially meet the daily recommended nutrient intake (RNI) for pregnant and lactating women and children (6–11 m children and 12–23 m children). These groups were emphasized because of their high nutritional vulnerability in Bangladesh, and they are considered as target groups in most nutrition interventions [8,9]. The calculation also represented the relative difference in the nutrient composition and density (nutrient content per 100 g of raw fish) within the considered fish species. For every considered nutrient, we highlighted five fish species with the highest nutrient content based on the data extraction. For comparison, two reference fish were considered: Atlantic cod (*Gadus morhua* L.) and Thai pangas (*Pangasianodon hypophthalmus*). Atlantic cod is a marine fish and was considered following the previous reference of Byrd et al. (2021) [30] whereas Thai pangas is an imported or exotic fish that is widely consumed in Bangladesh.

Nutrient content in the food items might change during processing and cooking. On the other hand, digestion and absorption of nutrients depends on physiological condition of individuals and the presence of stimulating and inhibitory factors in the diet. For example, dietary phytates inhibit iron absorption while vitamin C catalyzes it. Therefore, our calculations don't indicate any individual dietary advice but represent potentially of the respective marine fish to meet the daily RNI of the selected nutrients for the target groups, including pregnant and lactating women, infants 6–11 months, and children 12–23 months [31]. Following a method that was previously used, a daily serving of 50 g fish for women and 25 g fish for children was considered during calculation [30,32].

In our calculation, we assumed 10% bioavailability for iron [31]. For pregnant women, the daily RNI of iron was calculated based on the FAO/WHO (2004) (31) recommended iron intake for women aged 19–50 years because the daily RNI of iron for pregnant women was not specified. However, our estimated value (29.4 mg iron) closely aligns with other reference values, including Institute of Medicine's recommendation (27 mg iron per day) and the Indian Council of Medical Research's (ICMR) recommendation (35 mg iron per day) for pregnant women [33,34]. The daily RNI of protein for children aged 12–23 months and for pregnant and lactating women were estimated based on ICMR (2011) [33]; however, this guideline did not specify the daily RNI of protein for infants aged 6–12 months. Therefore, median body weight of boys and girls at 9 months of age, which is the average and median value between 6 and 12 months, was considered and the average standard body weight at 9 months was calculated [35]. The estimated standard body weight was then multiplied by 1.69 to calculate the recommended daily protein intake [33]. For zinc, we considered moderate bioavailability [31]. Furthermore, for pregnant and lactating women, daily zinc requirement was estimated by averaging the requirements during each of the three trimesters and considering requirement during first 12 months of lactation, respectively. This calculation provided a daily requirement of 7.5 mg zinc for pregnant women and 8.5 mg zinc for lactating women. To estimate the daily RNI for calcium and vitamin A, FAO/WHO (2004) [31] recommendation was followed. Finally, FAO recommended daily 200 mg/d DHA intake for pregnant and lactating women, while the adequate daily DHA intake for children 6–23 months old was estimated at 10–12 mg per kg body weight per day [36]. Following Bogard et al. (2015) [32], however, we considered daily 110 mg DHA requirement for children aged 6–23 months. Note that this is the midpoint of the recommended range of DHA intakes based on the respective body weights of children at 7 months and 23 months of age at the 50th percentile [35].

## 3 Results

**Fig. 1:**
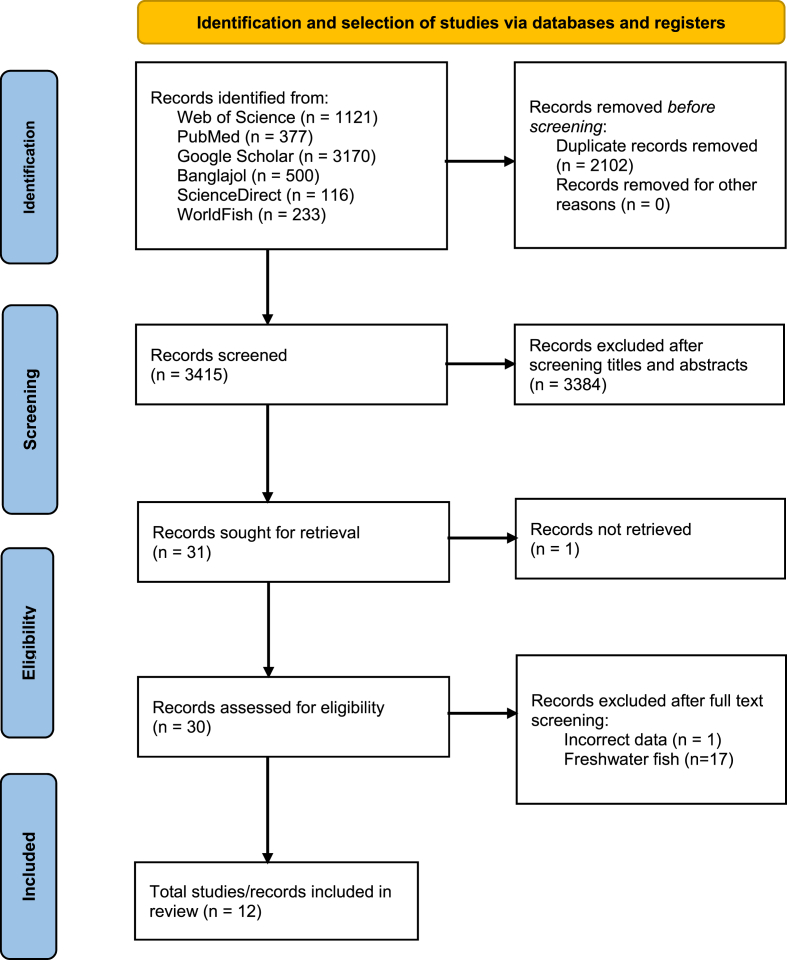
Selection process of the studies included in the review.

Through searching, 3463 articles or records were retrieved after excluding duplications and initial screening. For the next step, 3432 articles were excluded after screening the titles and the abstracts. A total of 31 articles were then given a full-text screening. We found one article/citation that could possibly met the inclusion criteria but was not available online [37]. Another article was excluded due to incorrect data because the sum of the proximate compositions (%) of the considered nutrients exceeded hundred and the units of measurements were not clarified. Finally, after full-text screening, 12 articles fulfilled the inclusion criteria and were finally considered for review (Fig. 1).

Table 1 shows the articles included in the review, number of fish species analyzed, sampling, and nutrients considered for analysis.

**Table 1:** Characteristics of the included studies.

Author	Number of fish species analyzed	Place of sample collection/sampling	Nutrients considered for analysis
Azam et al. (2004) [38]	10	Kuakata marine fish landing center	•Proximate composition: moisture, ash, protein, fat
Bhuiyan et al. (2006) [39]	3	Marine fish landing center in Chattogram	•Fatty acid composition: SFA, MUFA, PUFA, and n-3 and n-6 fatty acids
Bogard et al. (2015) [32]	8	Fish landing site in Cox's Bazar	•Macronutrients: energy, protein, fat, moisture, ash
			•Minerals: iron, zinc, calcium, iodine, selenium, phosphorus, magnesium, sodium, potassium, manganese, sulphur, copper
			•Vitamin: A^a^, D_2_, D_3,_ B_12,_ E and folate
			•Fatty acid profile: SFAs, UFAs, MUFAs, PUFAs
Hossain et al. (2014) [40]	1	Local fish market in Dhaka	•Proximate composition: moisture, protein, lipid, ash
			•Minerals: phosphorus, calcium, magnesium, sodium, potassium, zinc, iron, copper, manganese
			•Fatty acid profile: SFAs, UFAs, MUFAs, PUFAs
Mansur et al. (2018) [41]	5	Four samples from a fish market in Cox's Bazar and one from Mymensingh	•Proximate composition: Protein, lipid, ash, moisture
			•Heavy metal concentration: cadmium, chromium, copper, lead
Nordhagen et al. (2020) [42]	7	Bay of Bengal or deep sea (collected by EAF-Nansen program)	•Proximate composition: dry matter (%), protein, fat
			•Minerals: calcium, sodium, potassium, manganese, phosphorus, iodine, selenium, zinc, iron
			•Vitamins: A_1_, A_2_, B_12_, D
			•Fatty acid profile: SFAs, UFAs, MUFAs, PUFAs
Rahman et al. (2018) [43]	5	Fishery ghat (landing center) in Chittagong	•Amino acid profile
Rahman et al. (2018) [44]	5	Fishery ghat (landing center) in Chittagong	•Proximate composition: moisture, ash, lipid, protein, energy
			•Minerals and heavy metals: nickel, iron, zinc, cadmium, chromium, manganese, copper, calcium, magnesium, potassium, sodium, cobalt, lead
Shaheen et al. (2013) [22]	15	Stratified sampling plan based on the National Population Census Model	•Proximate composition: moisture, carbohydrate, protein, fat, ash, fiber, energy
			•Minerals: iron, zinc, calcium, iron, selenium, phosphorus, magnesium, sodium, potassium, magnesium, sulphur, copper
			•Vitamins: D, E, folate, A, B_1_, B_2_, B_3_, B_6_, C
Uddin et al. (2001) [45]	21	Marine fish landing center in Cox's Bazar	•Fat and cholesterol
Yusuf et al. (1993) [46]	12	Seafood shops in Dhaka	•Fatty acid profile: SFA, MUFA, n-3, n-6
Zaman et al. (2014) [47]	5	Local markets in Dhaka	•Proximate analysis: moisture, protein, fat, ash, energy
			•Minerals: iron, zinc, calcium, magnesium, sodium, potassium, manganese

a Vitamin A data presented in Bogard et al. (2015) [32] was published by Roos et al. (2001) [48].

We found 97 entries regarding the analysis of marine fish for any nutrient. In some cases, several researchers analyzed a single species. As a result, the total number of marine fish species analyzed for any nutrient ended up being 67. Uddin et al. (2001) [45] analyzed the maximum number of fish species [21] though only total fat and cholesterol were assessed. Bogard et al. (2015) [32] and Nordhagen et al. (2020) [42] analyzed the nutrients of the maximum number of categories, including proximate composition, minerals, vitamins, and a fatty acid profile. Bogard et al. (2015) (32) analyzed eight marine fish, while Nordhagen et al. (2020) [42] analyzed seven. In comparison, the Food Composition Table of Bangladesh represents nutrient composition of 15 marine fish, providing proximate composition and vitamins and minerals contents [22]. Hossain et al. (2014) [40] analyzed proximate composition, minerals, and fatty acid profile of *hilsa* (*Tenualosa ilisha*).

Table 2 represents the average nutrient content and the name of the fish species with the highest and the lowest nutritional value for relevant nutrients.

**Table 2:** Analyzed nutrients, average nutrient content, and name of fish species with the highest and the lowest nutrient content.

Nutrients (unit)	Number of entries or results^a^	Nutrient content per 100 g of edible raw marine fish^b^			Species with the highest value: Local name (*Scientific name*)	Species with the lowest value: Local name (*Scientific name*)	Reference (highest value, lowest value)
		Average value	Highest value	Lowest value			
**Proximate composition**							
Energy (kJ)	33	343.6	926	54.7	Hilsa (*Tenualosa ilisha*)	Loittya (*Harpadon nehereus*)	[22,44]
Moisture (g)	57	75.4	91.2	50	Loittya (*Harpadon nehereus*)	Hilsa (*Tenualosa ilisha*)	[40,42]
Protein (g)	57	16.8	25	7.4	Tuna/Maittya (*Euthynnus affinis*)	Loittya (*Harpadon nehereus*)	[22, 44]
Fat (g)	99	4.2	24.2	0.2	Hilsa (*Tenualosa ilisha*)	Toungsole/Kukurjib/Pata (*Cynoglossus bengalensis*)	[40, 45]
Ash (g)	50	2.2	8.4	0.7	Gang tengra (*Arius caelatus*)	Foli chanda (*Pampus argenteus*)	[32, 38]
**Minerals**							
Iron (mg)	42	2.13	8.6	0.2	Rupchanda (*Pampus chinensis*)	Loittya (*Harpadon nehereus*)	[42, 44]
Zinc (mg)	42	2.2	29.3	0.1	Poa (*Otolithoides pama*)	Kauwa (*Megalaspis cordyla*)	[42, 47]
Calcium (mg)	42	356.7	1900	13	Lal poa (*Johnius argentatus*)	Rupchanda/Foli chanda (*Pampus argenteus*)	[22, 32]
Iodine (μg)	15	39.2	160	6.9	Unicorn cod (*Bregmaceros mcclellandi*)	Parse (*Liza parsia*)	[32, 42]
Selenium (μg)	15	64.5	120	17	Puiya (*Benthosema fibulatum*)	Loittya (*Harpadon nehereus*)	[42]
Phosphorus (mg)	32	355.2	1000	110	Lal poa (*Pennahia argentata*)	Foli chanda (*Pampus argenteus*)	[32]
Magnesium (mg)	42	59.2	187.98	19	Koral/Vetkee (*Lates calcarifer*)	Loittya (*Harpadon nehereus*)	[42, 47]
Sodium (mg)	42	157.2	497.4	40	Loittya (*Harpadon nehereus*)	Fesha/Teli (*Setipinna taty*)	[22, 47]
Potassium (mg)	42	328.4	764	50	Parshe (*Liza parsia*)	Loittya (*Harpadon nehereus*)	[22, 47]
Manganese (mg)	20	0.2	1.1	0.01	Koral/Vetkee (*Lates calcarifer*)	Tailla (*Eleutheronema tetradactylum*)	[32, 47]
Sulphur (mg)	8	240	300	190	Tailla (*E. tetradactylum*)	Foli chanda (*Pampus argenteus*)	[32]
Copper (mg)	34	0.2	0.5	0.02	Churi (*Trichiurus haumela*)	Kalochanda (*Parastromateus niger*)	[32, 41]
**Vitamins**							
Vitamin B_12_ (μg)	14	4.7	15	0.5	Kauwa (*Megalaspis cordyla*)	Murbaila (*Platycephalus indicus*)	[32, 42]
Vitamin D (μg)	16	2.7	13	0.1	Tailla (*Eleutheronema tetradactylum*)	Foli chanda (*Pampus argenteus*)	[32]
Vitamin E (mg)	12	0.6	2.4	0.1	Koral/Vetkee (*Lates calcarifer*)	Murbaila (*Platycephalus indicus*)	[22, 32]
Folate (μg)	12	7.1	17	2.2	Poa (*Protonibea diacanthus*)	Murbaila (*Platycephalus indicus*)	[22, 32]
Vitamin A (μg)	14	43.8	288.7	7.3	Unicorn cod (*Bregmaceros mcclellandi*)	Loittya (*Harpadon nehereus*)	[42]
Thiamine (mg)	14	0.1	0.2	0.01	Tuna/Maittya (*Euthynnus affinis*)	Rupchanda, sada (*Pampus argenteus*)	[22]
Riboflavin (mg)	15	0.1	0.2	0.1	Lakkha/Gada (*Leptomelanosoma indicum*)	Fesha/Faishya (*Setipinna phasa*)	[22]
Niacin (mg)	15	5.7	19.3	0.7	Tuna/Maittya (*Euthynnus affinis*)	Koral/Vetkee (*Lates calcarifer*)	[22]
Vitamin B_6_ (mg)	12	0.3	0.9	0.1	Tuna/Maittya (*Euthynnus affinis*)	Koral/Vetkee (*Lates calcarifer*)	[22]
**Fatty acids**							
Total SFA (g)	25	2.3	12.6	0.1	Hilsa (*Tenualosa ilisha*)	Hilsa (muscle) (*Tenualosa ilisha*)	[40]
Total MUFA (g)	25	1.5	7.7	0.1	Hilsa (*Tenualosa ilisha*)	Loittya (*Harpadon nehereus*)	[40, 42]
Total n-3 (g)	25	0.6	2.99	0.02	Hilsa (*Tenualosa ilisha*)	Fesha/Teli (*Setipinna taty*)	[39,40]
Total n-6 (g)	25	0.2	0.7	0.02	Sting ray/Baga shaplapata (*Dasyatis uarnak*)	Rita (*Rita buchanani*)	[39,46]
EPA (mg)	10	340	1310	20	Hilsa (muscle) (*Tenualosa ilisha*)	Loittya (*Harpadon nehereus*)	[40,42]
DHA (mg)	10	408	1240	60	Hilsa (*Tenualosa ilisha*)	Loittya (*Harpadon nehereus*)	[40,42]

a Duplication (same species analyzed by several researchers) was considered.

b Values such as range, zero, not detected, missing values, trace were not considered while calculating the average.

Common nutrient analysis included proximate composition, minerals, vitamins, fatty acids, and amino acids. Proximate composition focused on energy, moisture, protein, fat, and ash. In total 12 minerals and nine vitamins were analyzed. The minerals were iron, zinc, calcium, iodine, selenium, phosphorus, magnesium, sodium, potassium, manganese, sulphur, and copper whereas the vitamins were A, B_1_, B_2_, B_3_, B_6_, B_12_, D, E, and folate. The fatty acid profile consisted of individual SFA, MUFA and PUFA, such as n-3 and n-6 fatty acids. We found the highest number of results for proximate composition analysis and the lowest for vitamin analysis. The findings show that different fish species are unique for different nutrient contents. For example, two different species of *poa* (*Pennahia argentata* and *Otolithoides pama*) were found with the highest content of total minerals (ash), iron, zinc, calcium, phosphorus, and folate. *Parshe* (*Liza parsia*) contains the highest amount of potassium, total n-6 fatty acids, EPA, and DHA. Meanwhile, *loittya* (*Harpadon nehereus*) was found to contain the lowest amount of energy, protein, iron, selenium, magnesium, potassium, vitamin A, total SFA, total MUFA, EPA and DHA. Rahman et al., 2018 [43] analyzed amino acid profile of five fishes and found they are rich source of histidine, isoleucine, phenylalanine, lysine, glutamic acid, aspartic acid, and arginine. Detailed data extraction, which includes species-wise nutrient contents, is provided in Supplementary file 1.

### 3.1 Potential contribution to address malnutrition

Based on current national and global nutrition context, we considered six nutrients: protein, iron, calcium, zinc, vitamin A and DHA. We then calculated what percentage of RNI one serving (50 g for adults and 25 g for children) of marine fish could meet for selected target groups (Supplementary file 2). For each nutrient, we considered top five species according to their nutrient content and compared them with those of Atlantic cod and Thai pangas.

### 3.2 Protein

Out of 97 entries, protein content was reported in 57 cases. Average protein content was 16.76 g per 100 g of edible raw marine fish. *Tuna* had the highest (25 g) amount of protein while *loittya* had the lowest (10 g) (Table 2). Besides tuna, fish such as hilsa, parshe or *bata* (*Mugil cephalus*), *chapila* (*Sardinella fimbriata*) and *kauwa* (*Megalaspis cordyla*) all have a good amount of protein, more than both Atlantic cod and Thai pangas. Note that *maricha* (*Dussumieria elopsoides*) has the same amount of protein (21 g per 100 g of edible raw marine fish) as both *chapila* and *kauwa* (Supplementary file 2).

**Fig. 2:**
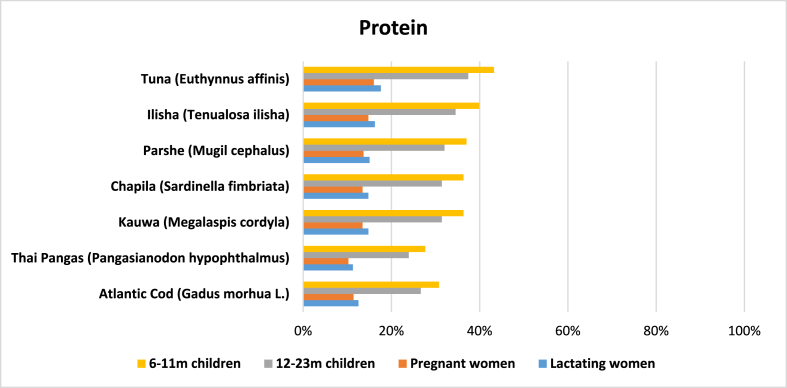
Potential contribution (%) of marine fish in Bangladesh to meet the RNI of protein for 6–11 m children, 12–23 m children, and pregnant and lactating women.

Protein deficiency is still a major nutritional problem in Bangladesh and other least developed countries. According to the Bangladesh demographic and health survey 2017–18, 8.4% of children under 5 years old are wasted [9]. Marine fish could be a good source of protein to meet this need. Our calculation shows that one serving of *tuna* could potentially meet 37.43% of the total daily protein requirement for children 12–23 months old and 17.61% for lactating women (Fig. 2).

### 3.3 Iron

**Fig. 3:**
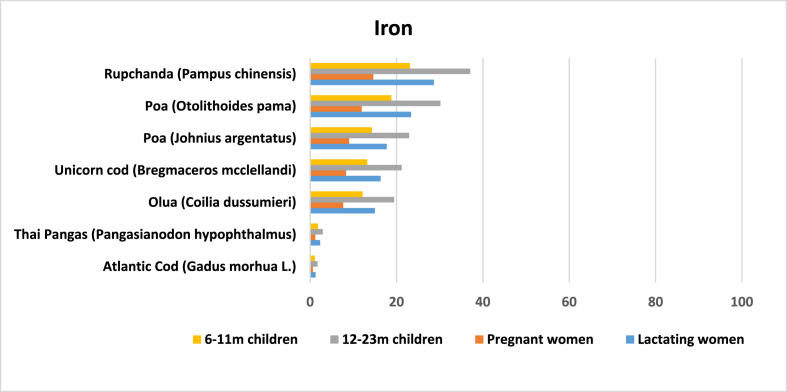
Potential contribution (%) of marine fish in Bangladesh to meet the RNI of iron for 6–11 m children, 12–23 m children, and pregnant and lactating women.

Out of 97 results, we found 42 entries for iron. Calculations show that the average amount of iron in marine fish is 2.13 mg per 100 g of edible raw marine fish. Rupchanda (*Pampus chinensis*) has the highest amount of iron (8.6 mg) while *loittya* has the lowest (0.2 mg). However, the amount of iron in *loittya* was reported in only two articles [42,47], and there was variation in the results. Zaman et al. (2014) [47] found 3.26 mg of iron per 100 g of edible raw marine fish, while Nordhagen et al. (2020) [42] found 0.2 mg. Apart from *rupchanda*, fish with comparatively high levels of iron are *poa* (*Otolithoides pama*), *lal poa* (*Johnius argentatus*), unicorn cod (*B. mcclellandi*) and *olua* (*Coilia dussumieri*). All five species have more iron than Atlantic cod and Thai pangas (Fig. 3).

Anemia from iron deficiency is a common nutritional problem among different age groups of people in Bangladesh. A national micronutrient survey showed that 10.7% of preschool children, 9.5% of school age children and 7.1% of NPNL women were iron deficient, while 33% of preschoolers and 50% of pregnant women were anemic [8]. Our calculation shows that one serving of *rupchanda* could potentially meet 37.07% of the daily iron requirement for children aged 12–23 months and 28.67% for lactating women.

### 3.4 Zinc

**Fig. 4:**
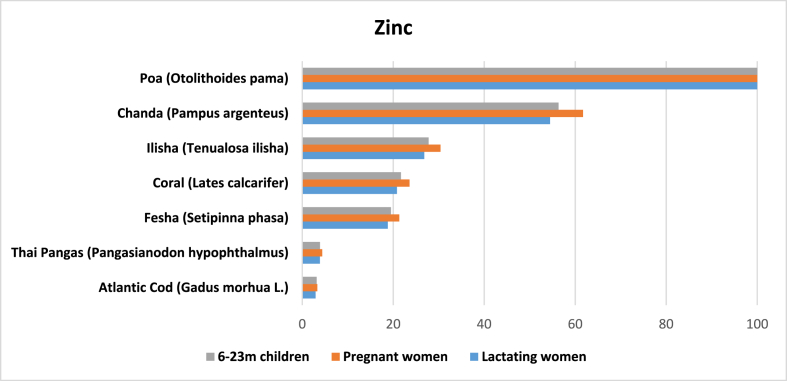
Potential contribution (%) of marine fish in Bangladesh to meet the RNI of zinc for 6–11 m children, 12–23 m children, and pregnant and lactating women.

A total of 42 entries with results were found for zinc. The data shows that the average amount of zinc in marine fish in Bangladesh is 2.2 mg per 100 g of edible raw marine fish. *Poa* (*Otolithoides pama*) has the highest (29.32 mg) and *kauwa* the lowest (0.1 mg) (Table 2). Other species with comparatively high in zinc are *Foli chanda*, *hilsa*, *koral/vetkee* and *fesha* or fhysha (*Setipinna phasa*). All are higher in zinc than Atlantic cod and Thai pangas (Fig. 4).

Although zinc is available in diverse food items, fish is one of the best sources of dietary zinc because of its high bioavailability. Despite being a leading fish producing country, zinc deficiency is still common in Bangladesh. It afflicts 44.6% of preschool children and 57.3% of NPNL women [8]. Our calculations indicated that one serving of *poa* could potentially meet 100% of the daily zinc requirement for the target populations.

### 3.5 Calcium

Calcium levels in marine fish were reported in 42 entries. The average amount of calcium was 356.71 mg per 100 g of edible raw marine fish. Based on available data, *lal poa* has the highest average amount (1900 mg) and *rupchanda* the lowest (13 mg) (Table 2). Marine fish species such as *kata phasa* (*Stolephorus tri*), *parshe*, *unicorn cod,* and *puiya* also had more calcium than the other species and substantially more than Atlantic cod and Thai pangas.

**Fig. 5:**
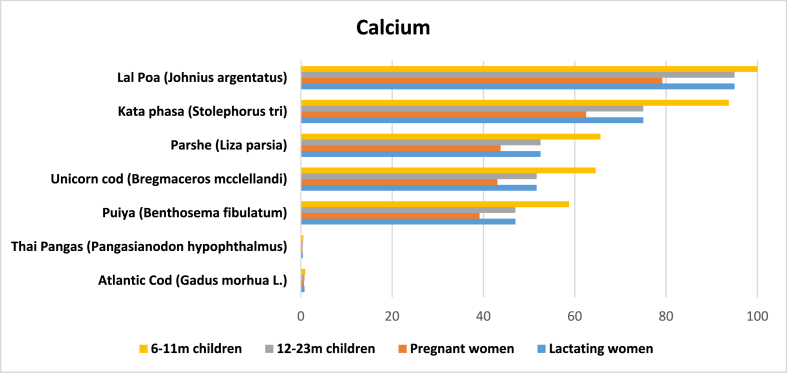
Potential contribution (%) of marine fish in Bangladesh to meet the RNI of calcium for 6–11 m children, 12–23 m children, and pregnant and lactating women.

Osteoporosis among adults, especially elderly women, from calcium deficiency is a common nutritional problem in Bangladesh. National data shows that 24.4% of preschool children, 17.6% of school-aged children and 26.3% of NPNL women suffer from calcium deficiency [8]. We calculated that one serving of *lal poa* could potentially meet almost entire daily calcium requirement for the target populations, while, on average, one serving of *kata phasa*, *parshe*, unicorn cod, and *puiya* could potentially meet almost half of their daily calcium requirements (Fig. 5).

### 3.6 Vitamin A

Three articles reported on the amount of vitamin A in marine fish [22,32,42]. Only 14 entries were found for vitamin A. The average amount of vitamin A was 43.75 μg per 100 g of edible raw marine fish. Unicorn cod had the highest average (288.7 μg) while *loittya* had the lowest (7.3 μg). Species such as *puiya*, *tuna*, *chompa* (*Scomberomorus commerson*) and *chapila* have comparatively higher levels than other species.

**Fig. 6:**
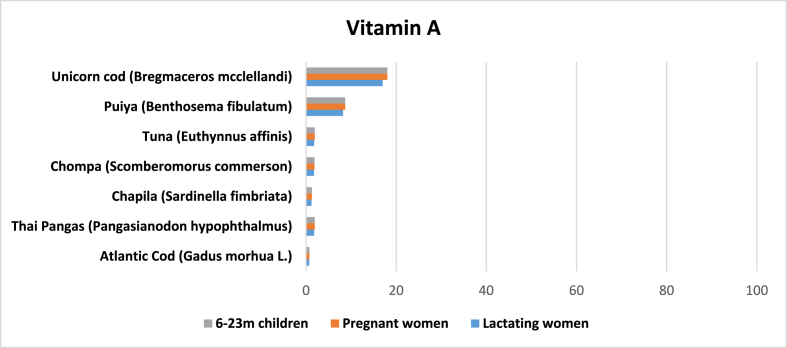
Potential contribution (%) of marine fish in Bangladesh to meet the RNI of vitamin A for 6–11 m children, 12–23 m children, and pregnant and lactating women.

Vitamin A is an essential nutrient for vision, growth, and immunity. In Bangladesh, although routine supplementation has significantly reduced vitamin A deficiency over the years, 20.5% of preschool children still suffer from sub-clinical vitamin A deficiency [8]. Vitamin A is widely present in colored vegetables, leaves, animal liver and fish. However, available data shows that analyzed marine fish were not a significant source of vitamin A. One serving of unicorn cod could meet 18.05% of the daily vitamin A requirement for children 6–23 months old, 18.04% for pregnant women and 16.98% for lactating women. Atlantic cod has the lowest amount of vitamin A among these species, while Thai pangas seems to have more than *chompa* and *chapila* (Fig. 6). However, the scarcity of evidence indicates that more research is needed to comment on the vitamin A content of marine fish in Bangladesh.

### 3.7 Docosahexaenoic acid (DHA)

Four articles reported the fatty acid profile of marine fish [32,40,42,46]. Out of 10 entries found for DHA, one contained the egg of *hilsa*. The average amount of DHA was 316 mg per 100 g of edible raw marine fish. *Hilsa* had the highest amount of DHA (693.9 mg) and *loittya* had the lowest (60 mg). Other species with high levels of DHA were *puiya*, *dom mach* (*Pentaprion longimanus*), *maricha* and *chapila*. All these marine fish species had higher DHA content than that of Thai pangas but lower than that of Atlantic cod.

**Fig. 7:**
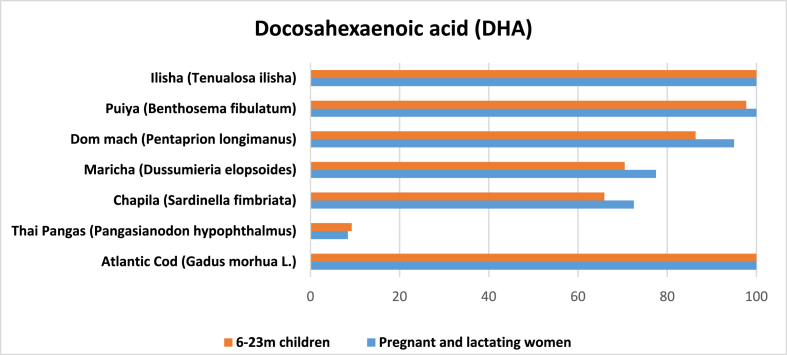
Potential contribution (%) of marine fish in Bangladesh to meet the RNI of DHA for 6–11 m children, 12–23 m children, and pregnant and lactating women.

Our calculation shows that one serving *hilsa* alone could potentially meet 100% of daily DHA requirement for the target population. Similarly, one serving of other four species—*puiya, dom mach, maricha*, and *chapila*—could potentially meet at least 60% of their daily DHA requirements (Fig. 7).

## 4 Discussion

### 4.1 Scarcity of data/literature exists

We found that 67 marine fish species (out of 97 entries) were analyzed for at least one nutrient. An updated list formulated by Habib et al. (2020) [49] showed 740 marine fish species in Bangladesh although 475 species are widely reported in literature [21,[50], [51], [52]]. In addition, Singha et al. (2019) [53] published an album of coastal and marine fish in Bangladesh in which 232 species were depicted. Considering 475 marine fish species, the nutritional values of only 12.8% of marine fish are known. Compared with global data, where 526 (25.7%) species were analyzed out of 2033 listed species [20], this is still quite low. Moreover, very few species were analyzed for a complete nutrient profile that includes proximate composition, vitamins, minerals, and fatty acids. Only two articles were found to represent a quality nutritional profile of 15 marine fish species [32,42]. This indicates a huge scarcity of data and literature in this area. Furthermore, analysis of some important nutrients, such as vitamin K, biotin, and molybdenum was not found in the included articles despite evidence showed these nutrients were identified in some fish and fish products [[54], [55], [56], [57]]. Commercially important fish such as *hilsa*, *loittya*, and *rupchanda* had been the research interest of many researchers; on the other hand, however, other available and affordable marine small fish such as *hicciri* (*Spratelloides gracilis*), *mola* (*Escualosa thoracata*), *kechki* (*Stolephorus commersonii*), *ganjana ilish* (*Tenualosa toli*), *choto churi* (*Eupleurogrammus muticus),* and *chewa* (*Pseudapocryptes elongates*) have not been analyzed yet.

### 4.2 Quality data should be prioritized

Nutrient composition of *loittya* was reported in three articles [41,42,47]. Although data did not highly vary for moisture and protein contents in these three articles, high variations were observed for fat, iron, zinc, and calcium contents. For example, zinc content measured by Zaman et al*.* (2014) [47] was 16 times higher than that of by Nordhagen et al. (2020) [42]. Similarly, Shaheen et al. (2013) [22] and Bogard et al. (2015) [32] measured *parshe* (*L. parsia*) but wide differences in lipid (2.4 times), ash (2 times), calcium (15.9 times), phosphorus (3 times), potassium (2.8 times), and copper (2.8 times) contents were observed. Differences in nutrient composition were also found for *hilsa, foli chanda,* and *koral/vetkee* (*L. calcarifar*) when estimated by different researchers.

Some common reasons behind the variation in results may be different methods used for nutrient analysis and differences in fish samples (size, age, seasons etc.) used for analysis [58]. For example, the big difference in zinc content in *loittya* samples as estimated by Zaman et al*.* (2014) [47] and Nordhagen et al. (2020) [42] could be due to the use of atomic absorption spectrophotometer and ICP-MS analysis, respectively, as analytical methods. Therefore, it is strongly recommended that researchers carefully report fish size, weight, maturity level, tissue processing, place of sample collection, duration between catch of the fish and nutrient analysis, and laboratory techniques used. To avoid further confusion and to maintain quality, duplicate samples may be analyzed.

### 4.3 Prioritize taxonomic identification

Taxonomic identification is important to understand the nutritional uniqueness of a specific species and to compare it with other species from the same family. In the reviewed articles, there were errors in taxonomic identifications. Nordhagen et al. (2020) [42] reported that there was no local name for unicorn cod, but a field-level investigation by WorldFish research team found that it is locally called *puiya*. Both *lal poa* and *poa* were reported as local names for *J. argentatus* [32,43]. On the other hand, *lal poa* is also reported as *Chrysochir aureus* [53] whereas others mentioned *lal poa* as *J. argentatus* or *P. argentata*. Therefore, it appears that the scientific name of *lal poa* has not been accurately reported in different articles. Yusuf et al. (1993) [46] reported that the local name of *Leignathus eguulus* as *rupchanda*, but *L. eguulus* is commonly and locally known as *poysa mach/chanda* (pony fish) according to our field observation. Furthermore, albacore tuna (*Thunnus germo*), chum salmon (*Oncorhynchus keta*), and Atlantic mackerel (*Scomber scombrus*) were reported as fish from Bangladesh [46]; however, investigation could be carried out to confirm if these species are from Bangladesh. Considering all of these, formulating a national database for marine fish representing their local names (with recommended spelling in both Bengali and English), common names (English), scientific names, picture, habits, classifications, zone of habitant (pelagic, mesopelagic, and demersal), length, weight and color at different life stages, and nutritional values from the available data could be a consideration.

### 4.4 Pelagic small fish could be a window of opportunity

There are several reasons to consider marine pelagic small fish for addressing malnutrition. First, nutrient composition of pelagic small fish shows their high nutritional quality compared to other types of fish. Fig. 3, Fig. 4, Fig. 5, Fig. 6, Fig. 7 represent the five most nutritious fish considering their protein, iron, zinc, calcium, vitamin A, and DHA contents. Both pelagic and mesopelagic fish are among the top five in many of these categories. They are high in protein, vitamin A, and DHA. Reksten et al. (2020) [59] assessed the nutrient levels of some common marine fish (n = 19) in Sri Lanka. Their analysis showed that small fish were more nutritious than large fish in every category (protein, fat, total dry matter, fatty acids, vitamin A, vitamin B_12_, vitamin D, calcium, iodine, iron, magnesium, phosphorus, selenium, sodium, and zinc) except potassium. Their findings also showed that pelagic fish were comparatively more nutritious than demersal fish considering protein, fat, iron, iodine, selenium, and sodium contents. Second, small-scale artisanal fishers mainly catch pelagic small fish. The top fish for marine capture include anchovy and Atlantic herring [2]. As such, pelagic small fish are more available to catch than other fish species by the small-scale fishers who comprise the biggest proportion of fishers involved in global fishery production. Furthermore, small-scale fishery production, which is mostly associated with pelagic small fish catch, had been recognized with poverty alleviation and improving food security [60]. Third, small pelagic fish are affordable, making them easily accessible for low socioeconomic groups. We found that *poa*, a demersal fish, is highly nutritious and commercially important, but had high price which could be unaffordable for the poor. The WorldFish research team conducted market investigations during January–May 2021 and found that 1 kg of *poa* or *lal poa* costed BDT 300–400 (USD 3–5). In contrast, pelagic fish such as *olua, fesha/fhysha, chapila/sardines, hicciri*, and *choto churi* (*Eupleurogrammus muticus*) were more affordable with the price of BDT 80–160 (USD 1–2) per kg. Large fish such as *hilsa* and *koral* has also high price. Researchers in Malawi, Zambia, and much of Southern Africa have shown that pelagic small fish were the commonly consumed fish in their countries, especially among the poor [61,62]. Fourth, certain production methods demonstrated that small pelagic fish were better and more sustainable for production than other species [[63], [64], [65], [66]], which means that production of pelagic small fish would be cost-effective. Fifth, there was evidence that low-cost pelagic small fish had been utilized to address malnutrition through dietary enrichment and using these fish in nutrition programs [67]. Therefore, accumulating records on availability, accessibility, affordability, nutritional value, preference, usability, and cost-effectiveness showed that low-cost but nutritious pelagic small fish could be useful to address common nutritional deficiencies in Bangladesh, if collected and utilized properly.

### 4.5 Food safety issues

Researchers analyzed the levels of heavy metals and assessed other health risks in marine fish. A recent study conducted by Reksten et al*.* (2021) [68] found that small marine fish from Bangladesh and Sri Lanka had higher levels of cadmium, arsenic, and lead than large fish, though large fish had higher mercury levels. However, they concluded that studied marine fish posed no health risks to adults and children when consumed in the recommended amounts. In addition, trace elements and heavy metals in the marine fish of Bangladesh were analyzed by Sharif et al. (1991) [69], Sharif et al. (1993) [70], and Khan et al. (1987) [71] and no health risk was identified. The findings suggested that marine fish in Bangladesh could contribute significant nutrients to the diet without posing health hazards. Recently, microplastic contamination in small fish, especially in the gut, has become a growing concern, though there is no strong evidence that food safety level is being threatened. However, considering all the potential hazards, further research is recommended to periodically estimate the risk-benefit ratio of fish consumption and provide dietary recommendation based on risk-benefit assessment [72,73]. We also suggest proper food handling and hygienic processing to avoid any potential health risks [58,74].

### 4.6 Not all commercially important fish are nutritious

According to DoF (2019) [4], species such as *loittya, churi, poa*, and *koral* are considered commercially important. *Loittya* is affordable and has high consumer preference, but our findings showed it had lower nutritional quality than other low-cost fish species (Table 2). For example, *loittya* had lower levels of energy, protein, iron, selenium, potassium, vitamin A, total SFA, total MUFA, EPA, and DHA than other species reported in this review. Our findings showed *poa* is nutritious but had low affordability. Results also showed that *churi* and *koral* are not as nutritious as other species that are not commercially important, such as small pelagic fish (Fig. 2, Fig. 3, Fig. 4, Fig. 5, Fig. 6, Fig. 7).

### 4.7 Comparison with other studies

Under the EAF-Nansen program, Reksten et al. (2020) [75] analyzed five marine fish from Angola, including two species of sardine (*Sardinella aurita* and *Sardinella maderensis*). Comparing the nutritional quality of sardine (*S. fimbriata*) from Bangladesh with those from Angola, results showed they have equal amounts of protein but *S. fimbriata* was higher in calcium, zinc, iron, iodine, and selenium than both *S. aurita* and *S. maderensis*.

Fernandes et al. (2014) [76] analyzed marine sardines from Brazil. It was observed *S. fimbriata* from Bangladesh was higher in protein but lower in fat than sardines from Brazil. Mohanty et al. (2017) [77] reported the nutrient value of *H. nehereus* from India, and the findings did not highly vary with those of Bangladesh. Lilly et al. (2017) [78] analyzed another species of sardine (*Sardinella albella)* from India, and the findings are closely similar with that of *S. fimbriata* from Bangladesh. Comparing with Osman et al. (2001) [79] regarding the content of fat content (2.79 g/100 g) of black pomfret from Malaysia, we found that black pomfret (*P. niger*) in Bangladesh was slightly high in fat content (3.7 g/100 g). Recently, Reksten et al. (2020) [59] analyzed the nutrient value of 19 marine fish from Sri Lanka. Their findings did not highly vary with those of the average nutrient contents of marine fish in Bangladesh.

### 4.8 Freshwater versus marine water fish

Bogard et al. (2015) [32] analyzed the nutrient composition of several small indigenous fish species (n = 30), major carps (n = 3), and introduced fish species (n = 8) in Bangladesh. Comparing results, marine fish in Bangladesh were higher in average protein (17.67 g/100 g) than small indigenous species (16.4 g/100 g), major carps (17.33 g/100 g), and introduced fish (17.54 g/100 g). Marine fish were also higher in iron (1.75 mg/100 g) than both major carps (1.44 mg/100 g) and introduced fish (1.71 mg/100 g). Average zinc content (2.25 mg/100 g) in marine fish was also higher than that of both major carps (1.2 mg/100 g) and introduced fish (1.33 mg/100 g). Regarding average calcium content, marine fish were higher (388.42 mg/100 g) than introduced fish (193.32 mg/100 g) but lower than small indigenous species (783.3 mg/100 g) and major carps (407 mg/100 g). Small indigenous species had higher levels of vitamin A (302.9 μg/100 g, n = 27) than marine fish (43.75 μg/100 g) (Table 2). *Mola*, an indigenous small fish, was more nutritious than marine fish in terms of energy, fat, iron, zinc, calcium, and vitamin A content [32].

### 4.9 Further scope

Despite progress in socioeconomic and nutritional indicators in previous decades, malnutrition is still a big public health problem in Bangladesh. Marine fish, especially pelagic small fish, could potentially address common nutritional deficiencies in the country. Previous studies showed that *mola,* an indigenous small fish, could potentially improve the status of iron and vitamin A among children in Bangladesh [80,81]. Similar studies could be carried out to observe the nutritional efficacy of some pelagic small fish.

Despite high nutritional values, small fish are considered difficult, due to presence of bones, for children to eat using traditional cooking methods. Considering this difficulty, introducing fish powder into meals could be a potential solution [82,83]. Including fish and fish-based products such as smashed fish, powdered fish, and fish chutney into the diet of children was recommended as a potential solution to address micronutrient deficiencies [84].

### 4.10 Limitations

There are some limitations of the evidence considered in the review. We found there was lack of high precision in the results when a single species was analyzed by different researchers. Therefore, when the findings from different studies is compared, the inference might not be accurate. Complete nutrient profile, for example proximate composition, vitamins, minerals, and fatty acids, was available only for few species. Therefore, further analysis of new species in the future might impact the average values (Table 2) in any direction. There are also some limitations in the review process. Due to the nature of the review and lack of available quality assessment tools suitable for the included articles, quality appraisal was not carried out.

### 4.11 Recommendations

More fish species should be analyzed to observe their nutrient composition. Analysis should emphasize complete nutrient profiling (proximate composition, vitamins, minerals, fatty acids, and amino acids) rather than estimating few nutrients. In addition, taxonomists should properly identify samples, in terms of local, common, and scientific names. Sampling should be carefully reported with data regarding the length and weight of species, maturity level, place of collection, tissue processing, duration between fish catch and nutrient analysis, and laboratory techniques used. A national database that includes the local name (with recommended spellings in both Bengali and English), common name, scientific name, picture, classification, and nature of niche (pelagic, mesopelagic, and demersal) could be created. Small marine pelagic fish, maximum 25 cm in length, could be considered for addressing nutritional deficiencies through proper interventions.

## 5 Conclusion

According to available data, marine fish are nutritious and have potential to address malnutrition due to protein, iron, zinc, calcium, and DHA deficiencies. Marine pelagic small fish are generally more nutritious than other fish; therefore, they could be effectively utilized to address malnutrition. Considering iron, zinc, and vitamin A content, marine fish are more nutritious than major and exotic carp, pangas, catfish, and tilapia but are less nutritious than inland indigenous small fish species. Literature is scarce regarding the nutritional quality of marine fish in Bangladesh, so it is suggested that more marine fish be analyzed. Variations in nutrient composition was observed when a specific species was analyzed and reported by several researchers; therefore, quality research with a special focus on analyzing vitamins, minerals, and heavy metals is recommended.

## Author contribution statement

All authors listed have significantly contributed to the development and the writing of this article.

## Funding statement

This research did not receive any specific grant from funding agencies in the public, commercial, or not-for-profit sectors.

## Data availability statement

Data included in article/supp. material/referenced in article.

## Declaration of interest's statement

The authors declare no competing interests.

## Appendix A Supplementary data

Data extraction file Data extraction file

Calculation of RNI Calculation of RNI
